# Still Water

**Published:** 1875-11

**Authors:** 


STILL WATER.

He wrote and wrote, but could not make a name:
Then cursed his fate and, called the world to blame—
The world, that knew not genius when it came.

"The world," he cried, "that crowns us in a night
For nothing; but condemns us, wrong or right,
Rather for sheer indifference than for spite."

One of his friends would slyly smile to hear;
"Ah! second-hand Bryonics!" one would sneer.
One said, "Give over." One said, "Persevere."

One said but little, though she thought and thought,
Through the long weeks and all the work they brought,
While the wife toiled and while the mother taught.

There went a story that he might have wed
An heiress, this poor scribbler for his bread,
But took a little meek-eyed girl instead.

A little meek-eyed girl without a cent,
Who scarcely knew what half his writings meant—
Loved him reveringly, and was content.

And now she mused and mused upon a way
To brighten his dull face again. One day
Her slender hand along his shoulder lay.

"Write this!" and then she told him what to write
In just a few fleet words, and stole from sight
With smiling lips, but with a look of fright.

He laughed at first, yet in a little space
The languid laughter died from out his face,
And left mute meditation in its place

If I Mistake not, it was this same year
That suddenly men knew him, far and near,
As having won the world's capricious ear.

And she? Why, if she had not seen so plain
How soon the laurels cured his longing pain,
She might have held them even in mild disdain.

But now she blesses Fortune's kind decree—
Proud, glad through him, though still, for all we see,
The same small, meek-eyed wife she used to be,

—*Lippincott for November.*

Church Music.—A listener thus describes the performance of our popular church music, and the effect produced upon his own mind: "The solemn worship of God was introduced by a solo, 'Consider the Lilies,' performed by the leading singer of the choir, and gracefully accompanied by the organ. So far as the music was concerned, it was beautifully and faultlessly rendered. The effect upon my own mind, however, was anything but devotional. The singer commenced, 'Consider the lilies of the field,' &c., and when she came to the application, it ran thus; 'And yet I say unto you—that even Solomon in all his glory—was not arrayed —was not arrayed—like one of these—was not arrayed—(interlude by the organ)—was not arrayed—(interlude by the organ)—like one of these; and then she went back again, and asserated in the most emphatic manner, 'I say unto you that even Solomon in all his glory was not arrayed — was not arrayed—was not arrayed—(pause,) until I began to despair for poor Solomon, lest he should never get the very first of his garments on. There was yet another piece of church (not sacred) music, in which the soprano led off with the announcement, 'I will wash;' and then came the contralto, 'I will wash;' and then the tenor, 'I will wash;' and then from the profoundest depths comes up the gutteral of the basso, saying also 'I will wash;' and last of all they stride in together, crying out in concert, 'I will wash.' No one could imagine that in this singular and oft-repeated announcement of an intended ablution, was rendered a sacred song for the spiritual edification of a Christian congregation of those solemn words of the Psalmist, "I will wash mine hands in innocency, and so will I compass thine altar, O Lord.— [ Brainard's Musical Work.

